# Portable, low-cost, desktop microscope

**DOI:** 10.1016/j.ohx.2024.e00593

**Published:** 2024-10-09

**Authors:** Vincent Salvadori, Daniel Fäh, Sarina Flühler, Jan Wandeler, Maria J. Jacome, Adrian Koller, Marcel Egli, Simon L. Wuest

**Affiliations:** a Lucerne School of Engineering and Architecture, Institute of Medical Engineering, Space Biology Group, Hergiswil, Switzerland; b Lucerne School of Engineering and Architecture, Institute of Mechanical Engineering and Energy Technology, Horw, Switzerland

**Keywords:** Microscope, Raspberry Pi, Camera, Portable, Low-cost

## Abstract

Light microscopes became essential tools in everyday lab work a long time ago. However, most commercial microscopes are costly, and they are often bulky and heavy. Therefore, microscopes are rarely seen in mobile applications or used by interested amateurs. Here, we present an affordable, portable single-lens microscope. It essentially uses a Raspberry Pi single-board computer, a camera, a touchscreen display, and an LED ring at its core. Apart from brightfield microscopy, contrast-enhancing methods by oblique, dark-field, and Rheinberg illumination are possible, as well. The microscope is ideal for applications that do not require high-end optical components. Due to its low cost and flexible use, it is also suitable for hands-on experiences of the fascinating world not visible by the human eye.

## Graphical abstract

**Figure:**
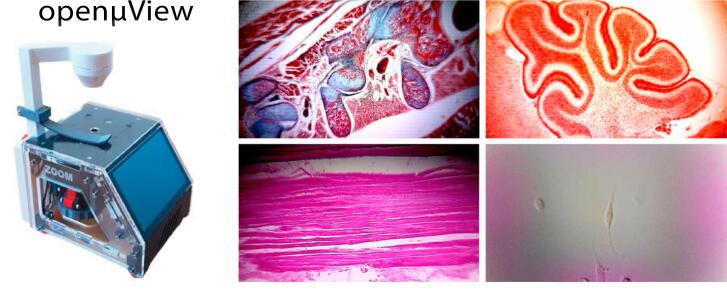

## 1 Specifications table

**Table:** 

Hardware name	open*µ*View (openMicroView)
Subject area	Biological sciences (e.g., microbiology and biochemistry) Educational tools and open-source alternatives to existing infrastructure General
Hardware type	Imaging tool
Closest commercial analog	Transmitted-light microscope
Open-source license	Creative Commons BY 4.0 (CC BY 4.0)
Cost of hardware	247 USD
Source file repository	https://doi.org/10.17632/p4v2kfp3js.1

## 2 Hardware in context

Simple transmission-light microscopes were introduced in the 16^th^ century [1]. Since then, they have continuously improved and have been responsible for countless scientific discoveries. A long time ago, light microscopes became essential tools for everyday lab work. Apart from their necessity in science and engineering, microscopes also open doors to the fascinating microscopic world not visible to the human eye. Most commercial microscopes are well manufactured, using expensive optical components. Furthermore, standard lab microscopes are often bulky and heavy and are therefore not suitable for mobile uses. In the past few years, several open-source, low-cost microscopes were published [2] which make use of commercial off-the-shelf products, including smart phones [3], plastic building blocks [4] and Raspberry Pi cameras [5], [6], [7]. Here, we present a simple, portable, low-cost single-lens microscope. It uses a Raspberry Pi single-board computer, a Raspberry Pi camera, a touchscreen display, and an LED ring at its core. The microscope is useful for field work in which portable microscopes are needed, as well as any lab applications that do not require the quality of high-end optical components. In addition, the low cost lowers the barriers to entry, making it useful for hands-on teaching and promoting curiosity about science.

## 3 Hardware description

**Fig. 1:**
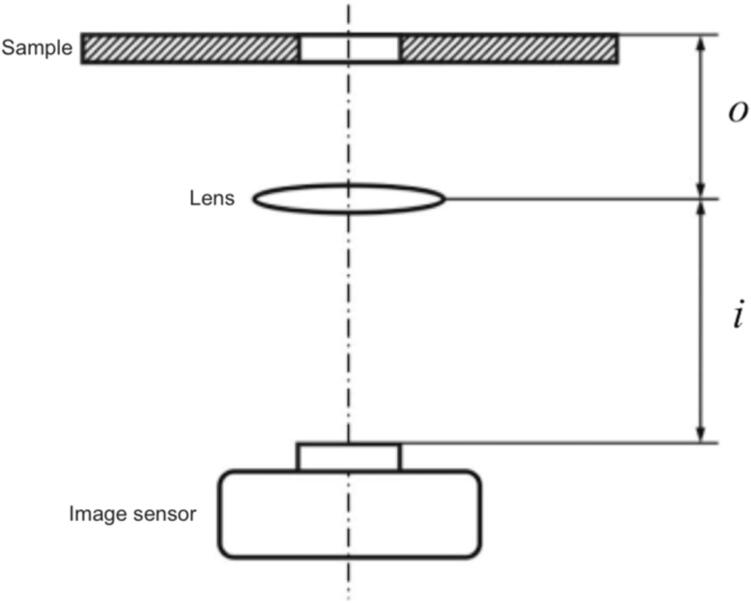
Schematic of a single-lens microscope. The optical magnification *M* of the sample onto the image sensor is the ratio of the distance *i* between the image sensor and the lens to the distance *o* between the lens and the sample.

The presented single-lens microscope uses a Raspberry Pi camera as the image sensor and its lens to magnify the sample’s image onto the sensor. The optical magnification *M* (x-fold) is the ratio of the distance *i* (mm) between the lens and the image sensor to the distance *o* (mm) between the object (in this case, the sample) and the lens (Fig. 1) [8]. The corresponding equation is:

$$M=\frac{i}{o}$$

To acquire a focused image, the following relationship must be respected:

$$\frac{1}{f}=\frac{1}{o}+\frac{1}{i}$$

where *f* (mm) is the focus length of the lens, which is 3.04  mm according to the camera’s data sheet.

**Fig. 2:**
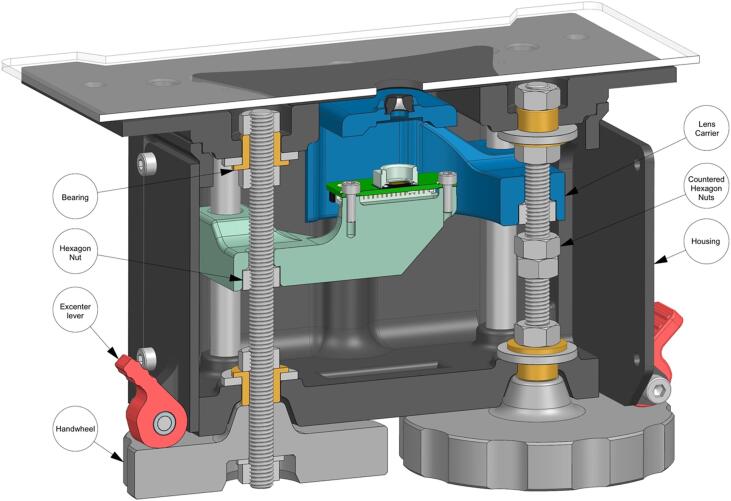
Cutaway view of the central mechanism to adjust magnification and focus. The sample’s image (positioned on the sample stage) is magnified by the lens into the camera. By turning two hand wheels, the user can linearly displace the lens and the camera, such that a focused image with the desired magnification is achieved.

The lens and the image sensor are both mounted on movable stages (Fig. 2), such that the user can adjust the distances o and i. By turning two hand wheels, which linearly displace the lens or the camera, the user can set the magnification and the focus. In the same way, the user can also adjust the magnification and focus to varying sample heights. Once the desired magnification and focus are achieved, the hand wheels can be clamped to avoid accidental displacements.

The camera is controlled by a Raspberry Pi single-board computer that can be conveniently operated via the Raspberry Pi touchscreen display. Sample illumination is realized with a four-color LED ring (red, green, blue, and white), which is also controllable via the Raspberry Pi computer. Custom-made software (written in Python) provides a graphical user interface (GUI) to fine tune illumination and camera settings and acquire images. It also allows time-lapse images. Acquired images are stored locally and can later be copied to external USB drives if desired.

**Fig. 3:**
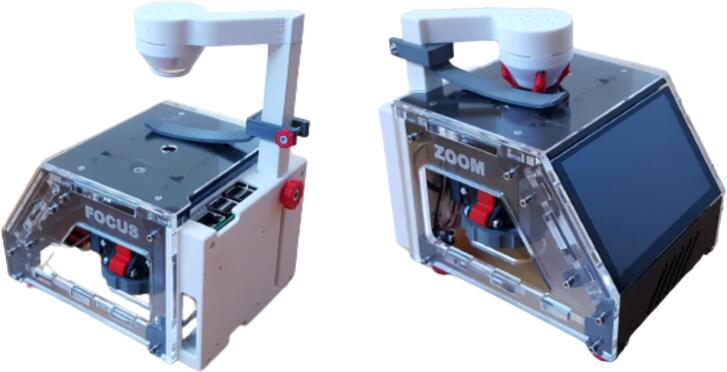
LED ring for sample illumination is mounted on a retractable lever such that the height can be adjusted for optimal imaging (left). For storage and transportation (right), a cover cap can be snapped in front of the LEDs, and the lever can be fully retracted to cover the lens and to protect it from dirt and dust.

The LED ring for illumination is mounted on a retractable lever (Fig. 3). This helps to adjust the illumination to achieve optimal contrast. Additionally, a magnetic cover cap can be snapped in front of the LEDs for storage and transportation. Then, the lever can be fully retracted so that the lens is covered to protect it from dirt and dust.

**Fig. 4:**
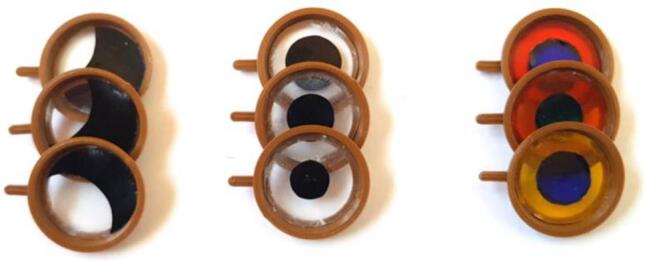
Image contrast can be enhanced by placing apertures or colored foils in front of the LEDs. Left: Kreutz apertures for oblique illumination. Center: aperture for dark-field illumination. Right: colored foils for Rheinberg illumination.

Thin biological samples with poor contrast, such as single cell cultures, are difficult to image with brightfield microscopy. However, image contrast can be enhanced by using simple apertures and colored foils, which are placed in front of the LEDs (Fig. 4). Depending on the sample, contrast can be greatly enhanced by using oblique [9], dark-field [10], or Rheinberg illumination [11]. Unused apertures can be stored below the screen (Fig. 3).

In oblique illumination, the sample is illuminated at an angle from the side [3], which creates a more contrasted brightness distribution with a one-sided relief. This can be achieved either by tilting the illumination to the side or by using a Kreutz aperture [12]. The latter has a light-tight foil with a crescentic opening on the side, such that the light falls at an angle onto the sample (Fig. 4, left). In comparison, in dark-field microscopy [10], the central area is obstructed such that no light can directly pass from the illumination to the objective lens (Fig. 4, center). Therefore, the background image generally appears dark. Part of the light from the outer illumination ring can be scattered by the sample such that it enters the objective lens. These light scattering structures then appear as bright objects in the image. Both techniques work well for almost transparent samples with poor contrast, such as thin biological samples. The Rheinberg illumination [11] uses the same principle as in dark-field illumination but uses two, often complementary, color filters. The central filter defines the background color, while the peripheral ring defines the color of the light scattered by the sample (Fig. 4, right). The Rheinberg illumination exploits the fact that the human eye can detect color differences better than differences in brightness.

## 4 Design files summary

**Table:** 

Design file name	File type	Open-source license	Location of the file
openMicroView_Case_DXF.zip	Laser cutting	CC BY 4.0	https://doi.org/10.17632/p4v2kfp3js.1
openMicroView_Case_STL.zip	3D printing	CC BY 4.0	https://doi.org/10.17632/p4v2kfp3js.1
openMicroView_Parts_STL.zip	3D printing	CC BY 4.0	https://doi.org/10.17632/p4v2kfp3js.1
openMicroView.stp	CAD	CC BY 4.0	https://doi.org/10.17632/p4v2kfp3js.1
openMicroView_Parts_NX.zip	NX CAD	CC BY 4.0	https://doi.org/10.17632/p4v2kfp3js.1
openMicroView_SW_v1.0.1.tar.gz	Software	GNU GPLv3	https://doi.org/10.17632/p4v2kfp3js.1

**Table:** 

File	Description
openMicroView_Case_DXF.zip	DXF-files for laser cut parts.
openMicroView_Case_STL.zip	STL-files for 3D-printed casing if laser cutter is not available.
openMicroView_Parts_STL.zip	STL-files for 3D-printed parts.
openMicroView.stp	3D model of the open*µ*View.
openMicroView_Parts_NX.zip	CAD in NX software (version 2007, released Dec. 2021). Assembly and part files.
openMicroView_SW_v1.0.1.tar.gz	Software package to operate microscope (Version 1.0.1).

## 5 Bill of materials

Components that are not commercially available first have to be 3D printed from ABS or PLA filaments. The major components of the housing are best manufactured from PMMA (polymethylmethacrylate) on a laser cutter. If no laser cutter is available, the parts can be 3D printed instead. Additionally, normal mechanical components (e.g., screws, nuts, and bearings) as well as commercial electronic products (EPs) are required. The light diffusor (E6) helps to achieve a homogeneous illumination and is a very thin 3D print. Therefore, preferably a white or at least light-colored material should be used. The microscope is divided in the main assembly categories: “housing” (H), “mechanical unit” (M), “electronic unit” (E), and “final assembly“ (F).

**Table:** 

Designator	Component	Number	Cost per unit − USD	Total cost − USD	Source of materials	Material type
H1	Angle	4	0.03	0.13	3D JAKE	ABS or PLA (3D print)
H2	Foot	4	0.03	0.13	3D JAKE	ABS or PLA (3D print)
H3	Cable clip	1	0.03	0.03	3D JAKE	ABS or PLA (3D print)
H4	Corner bracket left	1	0.07	0.07	3D JAKE	ABS or PLA (3D print)
H5	Corner bracket right	1	0.07	0.07	3D JAKE	ABS or PLA (3D print)
H6	Cyl. screw DIN912 M4x20	4	0.06	0.22	*^1^	Steel
H7	Cyl. screw DIN912 M2.5x08	18	0.06	1.01	*^1^	Steel
H8	Cyl. screw DIN912 M2.5x10	18	0.06	1.01	*^1^	Steel
H9	Double bracket left	1	0.07	0.07	3D JAKE	ABS or PLA (3D print)
*H*10	Double bracket right	1	0.07	0.07	3D JAKE	ABS or PLA (3D print)
*H*11	Filter clip	5	0.03	0.17	3D JAKE	ABS or PLA (3D print)
H12	Filter magazine	1	1.08	1.08	3D JAKE	ABS or PLA (3D print)
H13	Filter pan	>5	0.03	0.17	3D JAKE	ABS or PLA (3D print)
H14	Base plate	1	2.14	2.14	*^2^ or 3D JAKE	5 mm PMMA (laser cut) or ABS or PLA (3D print)
H15	Foot bracket	4	0.07	0.27	3D JAKE	ABS or PLA (3D print)
H16	Hexagon nut DIN934 M2.5	32	0.06	1.79	*^1^	Steel
H17	Hexagon nut DIN934 M4	4	0.06	0.22	*^1^	Steel
H18	Top plate	1	1.49	1.49	*^2^ or 3D JAKE	5 mm PMMA (laser cut) or ABS or PLA (3D print)
H19	Screen support	1	1.19	1.19	*^2^ or 3D JAKE	5 mm PMMA (laser cut) or ABS or PLA (3D print)
H20	Side wall left	1	1.88	1.88	*^2^ or 3D JAKE	5 mm PMMA (laser cut) or ABS or PLA (3D print)
H21	Side wall right	1	1.88	1.88	*^2^ or 3D JAKE	5 mm PMMA (laser cut) or ABS or PLA (3D print)
M1	Mechanics cover plate	1	1.31	1.31	3D JAKE	ABS or PLA (3D print)
M2	Cyl. screw DIN912 M2x06	6	0.06	0.34	*^1^	Steel
M3	Cyl. screw DIN912 M3x22	3	0.06	0.17	*^1^	Steel
M4	Cylinder pin DIN6325 Ø6x60	4	1.12	4.48	*^1^	Steel
M5	Disc DIN433-5	2	0.06	0.11	*^1^	Steel
M6	Disc DIN433-8	4	0.06	0.22	*^1^	Steel
M7	Excenter lever	2	0.03	0.07	3D JAKE	ABS or PLA (3D print)
M8	Handwheel	2	0.44	0.87	3D JAKE	ABS or PLA (3D print)
M9	Hexagon nut DIN934 M3	3	0.06	0.17	*^1^	Steel
M10	Hexagon nut DIN934 M5	13	0.06	0.73	*^1^	Steel
M11	Mechanics housing	1	2.92	2.92	3D JAKE	ABS or PLA (3D print)
M12	Camera carrier	1	0.30	0.30	3D JAKE	ABS or PLA (3D print)
M13	Joint plate	2	0.20	0.40	3D JAKE	ABS or PLA (3D print)
M14	Lens carrier	1	0.44	0.44	3D JAKE	ABS or PLA (3D print)
M15	Magnet Ø3x3	12	0.03	0.40	Brack	Metal
M16	Magnet holder	1	0.07	0.07	3D JAKE	ABS or PLA (3D print)
M17	Sample plate	1	1.14	1.14	*^2^ or 3D JAKE	1.5 mm PMMA (laser cut) or ABS or PLA (3D print)
M18	Plain bearing	4	0.03	0.13	3D JAKE	ABS or PLA (3D print)
M19	Sleeve	2	0.03	0.07	3D JAKE	ABS or PLA (3D print)
M20	Threaded rod M5x77mm	2	0.90	1.79	*^1^	Steel
E1	Clamping ring	1	0.13	0.13	3D JAKE	ABS or PLA (3D print)
E2	Lens cover	1	0.07	0.07	3D JAKE	ABS or PLA (3D print)
E3	Light support cover	1	0.29	0.29	*^2^ or 3D JAKE	3 mm PMMA (laser cut) or ABS or PLA (3D print)
E4	Cyl. screw DIN912 M5x12	1	0.11	0.11	*^1^	Steel
E5	Cyl. screw DIN912 M2.5x6	4	0.06	0.22	*^1^	Steel
E6	Diffusor	1	0.03	0.03	3D JAKE	ABS or PLA (3D print)
E7	Filter holder 1	1	0.24	0.24	3D JAKE	ABS or PLA (3D print)
E8	Filter holder 2	1	0.07	0.07	3D JAKE	ABS or PLA (3D print)
E9	Electronics housing	1	2.18	2.18	3D JAKE	ABS or PLA (3D print)
E10	Knurled knob M3	1	0.03	0.03	3D JAKE	ABS or PLA (3D print)
E11	Knurled knob M5	1	0.07	0.07	3D JAKE	ABS or PLA (3D print)
E12	Leaf spring	1	0.13	0.13	3D JAKE	ABS or PLA (3D print)
E13	Horizontal light support	1	0.60	0.60	3D JAKE	ABS or PLA (3D print)
E14	Vertical light support	1	0.50	0.50	3D JAKE	ABS or PLA (3D print)
F1	Ctsk. screw DIN7991 M2.5x12	10	0.06	0.56	*^1^	Steel
F2	Ctsk. screw DIN7991 M2.5x16	4	0.06	0.22	*^1^	Steel

**Table:** Commercial electronic products:

Designator	Component	Number	Cost per unit − USD	Total cost − USD	Source of materials	Manufacturer Part No.
EP1	Fan 25x25mm	1	4.93	4.93	BerryBase	RPI-FANKK
EP2	LED ring Adafruit NeoPixel Jewel 7x	1	11.09	11.09	Digi Key Distrelec Reichelt	1528–1610-ND 300–91-174 DEBO NP JEWEL
EP3	Lens biconvex Ø25 F = 44.5 mm	1	2.52	2.52	OPITEC	820,648
EP4	Raspberry Pi 3 Model B	1	35.22	35.22	Digi Key Distrelec	2648-SC0073-ND 301–35-058
EP5	Raspberry Pi Camera V2 (image sensor)	1	32.37	32.37	Digi Key Distrelec	2648-SC0023-ND 301–34-462
EP6 *^3^	Raspberry Pi Camera V2 lensShipped with camera (EP5)	1	−	−	−	−
EP7	Touch Screen DFRobot 5	1	72.69	72.69	Digi Key	1738–1453-ND
EP8	Ribbon cable, 300 mm (Camera Cable)	2	1.96	3.92	DigiKey	1528–2107-ND
	Memory Card, microSDHC, 32 GB	1	19.68	19.68	DigiKey Distrelec	4670-IUDD33K-032GR-ND 301–10-941
	Jump wires	1	5.71	5.71	DigiKey Mouser Reichelt JOY-IT	438-TW-FF-30C-ND 589-TW-FF-30C DEBO KABELSET5 RB-CB3-050

*^1^: Standardized machine components (e.g., screws) can be obtained from a local hardware store or supplier, such as Debrunner Acifer.

*^2^: PMMA (polymethylmethacrylate), also known as acrylic glass or by the trade name Plexiglas, can be obtained from a local hardware store or supplier.

*^3^: The lens is the original lens shipped with the camera. The lens has its own little housing with an aperture at the front end which helps to avoid a blurred image. The lens is screwed into the camera module, such that the focus can be adjusted by simply turning the lens. The lens (including its housing) can easily be separated from the camera with the provided tool.

To manufacture the apertures and filters, the following additional products will be required, depending on the filters desired.

**Table:** 

Designator	Component	Number	Cost per unit − USD	Total cost − USD	Source of materials	Manufacturer Part No.
A1	Transparent foil (e.g. Q-CONNECT, PVC binding covers, A4, 250 µm, KF24011)	2	0.38	0.76	*^4^ or Q-CONNECT	KF24011
A2	Black adhesive foil	1	8.46	8.46	*^4^ or d-c-fix	F2000111
A3	Colored window films	1	11.92	11.92	*^4^ or folia Bringmann	455,409
Total				247		

*^4^: Transparent, black, and colored foils can be obtained from a local stationery store or supplier.

## 6 Build instructions

In addition, the following material is required for assembly:

• Hex key set
• Drill Ø6 mm
• Flat-nose pliers (2x)
• Small metal saw
• Instant glue *
• File to deburr
• Side cutter
• Cable stripper
• Soldering tin
• Soldering iron
• Circle cutter
• Scissors or scalpel
• Permanent marker

*: We used a common ethyl cyanoacrylate based instant glue which had a thin dosing nozzle for precise application. However, the vapors from cyanoacrylate glue could deposit on optical elements and impair their function. Therefore, to glue the biconvex lens (EP3) into the “filter holder 1” E7 (see below), it is advisable to use two-component epoxy resin.

### 6.1 Mechanical assembly

#### 6.1.1 Housing

1. Press M2.5 nuts (H16) into “angles” H1 (4x; Fig. 5). If necessary, fix with glue.

**Fig. 5:**
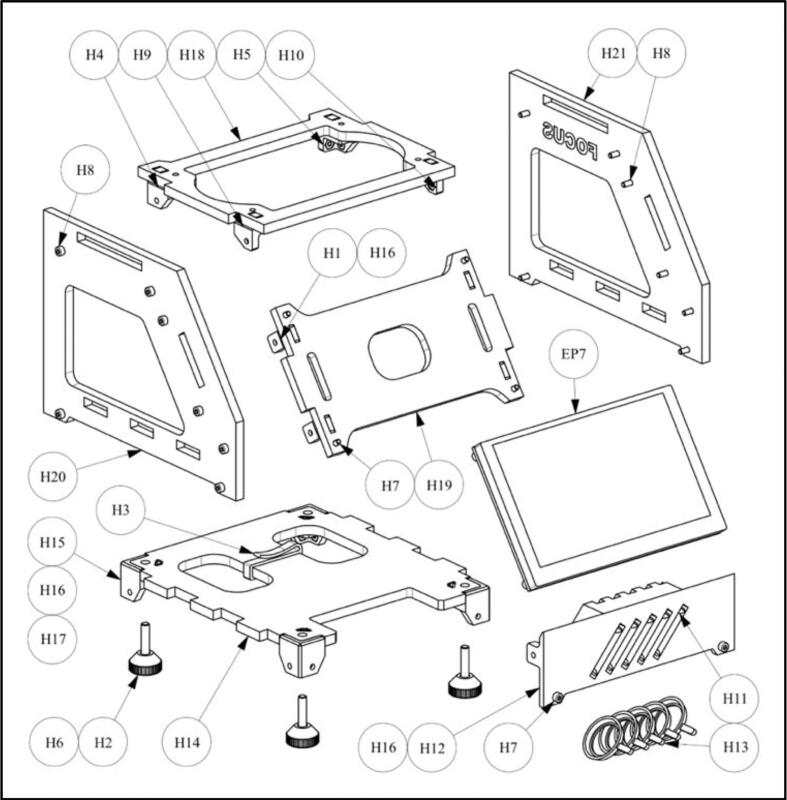
Schematic of housing assembly.
2. Press “angles” H1 into the “screen support” H19 (4x). If necessary, fix with glue.
3. Connect the ribbon cable (F3) to the touch screen (EP7) and guide it through the slot in the “screen support” H19.
4. Attach touch screen (EP7) with four M2.5x8 screws (H7) to the “screen support” H19.
5. Press M2.5 nuts (H16) into “filter magazine” H12 (2x). If necessary, fix with glue.
6. Glue “filter clips” H11 into “filter magazine” H12 (5x).
7. Press M2.5 nuts (H16) into “double bracket left” H9 (2x). If necessary, fix with glue.
8. Press M2.5 nuts (H16) into “double bracket right” H10 (2x). If necessary, fix with glue.
9. Press M2.5 nuts (H16) into “corner bracket left” H4 (3x). If necessary, fix with glue.
10. Press M2.5 nuts H16 into “corner bracket right” H5 (3x). If necessary, fix with glue.
11. Press “corner bracket left” H4, “corner bracket right” H5, “double bracket left” H9, and “double bracket right” H10 into “top plate” H18. If necessary, fix with glue.
12. Press M4 nut (H17, 1x) and M2.5 nuts (H16, 2x) into “foot bracket” H15 (4x). If necessary, fix with glue.
13. Press “foot bracket” H15 into “base plate” H14 (4x). If necessary, fix with glue.
14. Glue M4x20 screw (H6) to “foot” H2 with glue (4x).
15. Mount “foot” H2 to “base plate” H14 (4x).
16. Mount “cable clip” H3 to “base plate” H14.
17. Mount “filter magazine” H12 with two M2.5x8 screws (H7) to “base plate” H14.
18. Mount “base plate” H14 and “filter magazine” H12 to “side wall left” H20 using three M2.5x10 screws (H8).
19. Mount “screen support” H19 with two M2.5x10 screws (H8) to “side wall left” H20.
20. Clip the ribbon cable (F3) from the touch screen (EP7) into the “cable clip” H3.
21. Mount “top plate” H18 with two M2.5x10 screws (H8) to the “side wall left” H20.
22. Mount the “side wall right” H21 with seven M2.5x10 screws (H8) to the assembly H14, H12, H19, and H18.

#### 6.1.2 Mechanical unit

1. Press M2.5 nuts (H16) into “mechanics housing” M11 (8x; Fig. 6). If necessary, fix with glue.

**Fig. 6:**
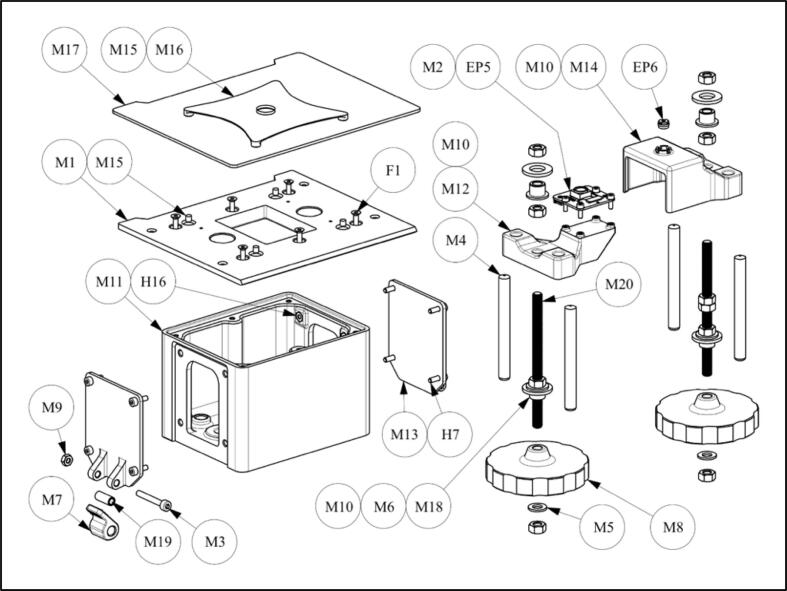
Schematic of mechanical unit assembly.
2. Insert Ø6x60 cylindrical pins (pos. M4) into “mechanics housing” M11 (4x). The holes of Ø6 mm in the “mechanics housing” M11 may need to be enlarged (4x). The cylindrical pins (pos. M4, 4x) should be able to be mountable with little effort.
3. Glue four magnets Ø3x3 (M15, 4x) into “mechanics cover plate” M1. Ensure that the magnet’s poles are oriented the same way!
4. Glue four magnets Ø3x3 (M15, 4x) into “magnet holder” M16. Ensure that the magnets are oriented such that the “magnet holder” M16 can be snapped onto the “mechanics cover plate” M1. The magnets should attract (not repel) the two components.
5. Glue “magnet holder” M16 to “sample plate” M17.
6. Press an M5 nut (pos. M10) into “image sensor carrier” M12. Ensure that the nut is leveled! If necessary, fix with glue.
7. Mount camera (EP5) with four M2x6 screws (pos. M2) onto the “image sensor carrier” M12.
8. Press an M5 nut (pos. M10) into the “lens carrier” M14. Ensure that the nut is leveled! If necessary, fix with glue.
9. Remove the lens (including its housing; EP6) from the camera using the provided tool. Then carefully press it into the “lens carrier” M14.
10. If required, cut two M5 threaded rods to 77  mm length and deburr.
11. Mount four M5 nuts (pos. M10) on the two threaded rods M5x77 mm (pos. M20), as indicated in the sketch Fig. 7.

**Fig. 7:**
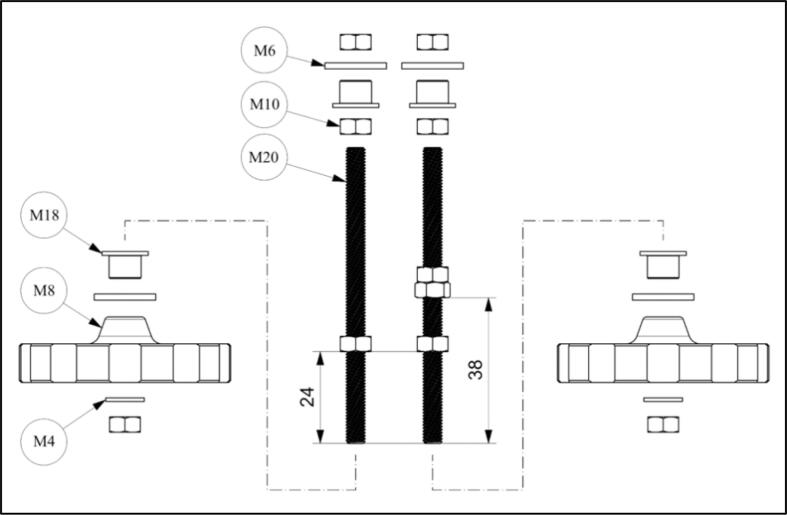
Schematic of threaded rods and handwheels assembly.
12. Push a “plain bearing” M18 and a disc (ID = 8.4  mm, OD = 15  mm, h = 1.6  mm; pos. M6) from below into each of the threaded rods (pos. M20) according to the sketch (2x; Fig. 7). Do not attach the remaining positions yet!
13. Insert the threaded rods (pos. M20) from above into the “mechanics housing” M11 (2x). Make sure to mount the rods on the appropriate sides!
14. Mount the two “handwheels” M8, using a disc (ID = 5.3  mm, OD = 9 mm, h = 1 mm; pos. M4) and an M5 nut (pos. M10), on the threaded rods (pos. M20) from below (2x).
15. Guide the ribbon cable through the slot in the “mechanics housing” M11 and connect it to the camera (EP5).
16. Slide the “image sensor carrier” M12 from above onto the left threaded rod (pos. M20, left) and move it all the way down by turning the “handwheel” M8. The holes of Ø6 mm in the “image sensor carrier” M12 may need to be enlarged (2x). The “image sensor carrier” M12 should be able to slide along the cylindrical pins (pos. M4) without much friction.
17. Slide the “lens carrier” M14 from above onto the right threaded rod (pos. M20, right) and move it all the way down by turning the “handwheel” M8. The holes of Ø6 mm in the “lens carrier” M14 may need to be enlarged (2x). The “lens carrier” M14 should be able to slide along the cylindrical pins (pos. M4) without much friction.
18. Mount an M5 nut (pos. M10), a “plain bearing” M18, and a disc (ID 8.4  mm; pos. M6) from above onto each of the threaded rods (pos. M20) according to the sketch (Fig. 7), and move them all the way down (2x).
19. Press the “mechanics cover plate” M1 onto the four Ø6x60 cylindrical pins (pos. M4) and fasten it to the “mechanics housing” M11 using six M2.5 countersunk screws (F1). The holes of Ø6 mm in the “mechanics cover plate” M1 may need to be enlarged (4x). The “mechanics cover plate” M1 should be able to be installed with little effort.
20. Move the M5 nut (pos. M10), the “plain bearing” M18, and the disc (pos. M6) upwards from the outside until the disc (pos. M6) is in contact with the “mechanics cover plate” M1. Do this for both sides. Do not tighten!
21. Mount an M5 nut (pos. M10) onto the threaded rods (pos. M20) from above on both sides and tighten (2x).
22. Insert the “sleeves” M19 into the “excenter levers” M7 (2x).
23. Position the “sleeves” M19 and the “excenter lever” M7 in the “joint plates” M13 and fasten with an M3x22 screw (pos. M3) and an M3 nut (pos. M9) on both sides (2x).
24. Fasten both “joint plates” M13 each with four 2.5x8 screws (H7) to the “mechanics housing” M11 (2x).

#### 6.1.3 Electronic unit

1. Glue the fan (EP1) onto the Raspberry Pi (EP4; Fig. 8).

**Fig. 8:**
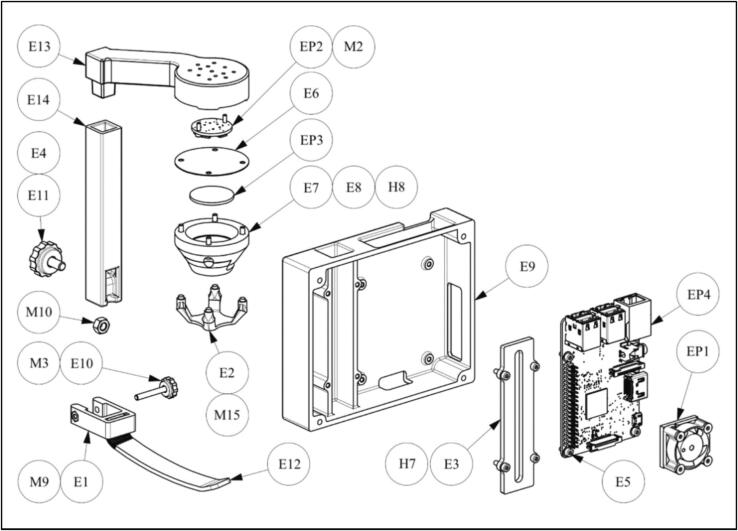
Schematic of electronic unit assembly.
2. Connect the fan’s black wire to pin 6 (ground) and the red wire to pin 4 (5 VDC; Fig. 9). Please note: In this configuration, the fan is always running as soon as the Raspberry Pi is powered (even when it was shut down previously). For a more sophisticated solution, an alternative GPIO can be used. Please also see “6.6.2 Fan operation” for more details.

**Fig. 9:**
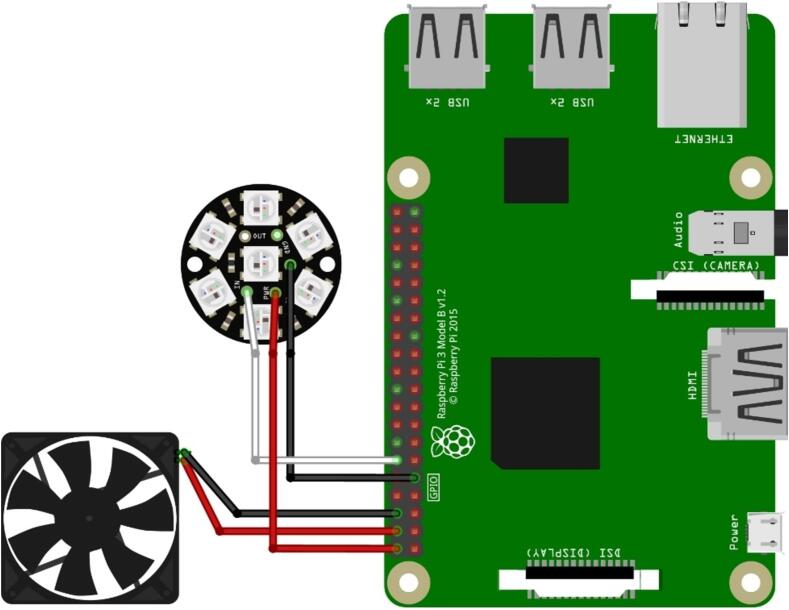
Connection of the LEDs and the fan to the Raspberry Pi.
3. Fasten the Raspberry Pi (EP4) with four M2.5x6 screws (E5) on the “electronic housing” E9.
4. Solder three wires (ca. 30  cm or longer) to the LED ring (EP2): Black wire goes to “GND” (ground); red wire on “5V DC Power”; white wire goes on “Data Input”. Premade jumper wires, such as listed in the bill of materials, are an easy way to connect to the Raspberry Pi. But wires may also made from scratch if the required tools are available.
5. Attach the LED ring (EP2) with two M2x6 screws (pos. M2) to the “horizontal light support” E13 and guide the cable through the “horizontal light support” E13.
6. Join “filter holder 1” E7 and “filter holder 2” E8 with glue.
7. Carefully glue the biconvex lens (Ø25; EP3) into the “filter holder 1” E7.
8. Attach the “filter holder 1” E7 and the “diffusor” E6 to the “horizontal light support” E13 using four M2.5x10 screws (H8).
9. Guide the LED cable through the “vertical light support” E14 and insert the “horizontal light support” E13 into the “vertical light support” E14.
10. Press a M3 nut (pos. M9) into the “clamping ring” E1. If necessary, fix with glue.
11. Glue “leaf spring” E12 to “clamping ring” E1 with glue.
12. Glue an M3x22 screw (pos. M3) into the “knurled knob M3” E10.
13. Slide the “clamping ring” E1 on the “vertical light support” E14 and clamp with the “knurled knob M3” E10.
14. Glue an M5x12 screw (E4) into the “knurled knob M5” E11.
15. Position an M5 nut (pos. M10) in the “vertical light support” E14.
16. Slide the “vertical light support” E14 into the “electronics housing” E9 and clamp it with “knurled knob M5” E11.
17. Guide the LED cable through the “light support cover” E3 and connect it to the Raspberry Pi (EP4; Fig. 9). Black wire goes on pin 9 (ground); red wire on pin 2 (5 VDC); white wire goes on pin 12 (GPIO 18).
18. Attach the “light support cover” E3 to the “electronics housing” E9 using four M2.5x8 screws (H7).
19. Glue four magnets Ø3x3 (M15, 4x) to the “lens cover” E2.

#### 6.1.4 Final assembly

1. Insert the mechanical unit into the housing from above and secure it with four M2.5x16 countersunk screws (F2; Fig. 10).

**Fig. 10:**
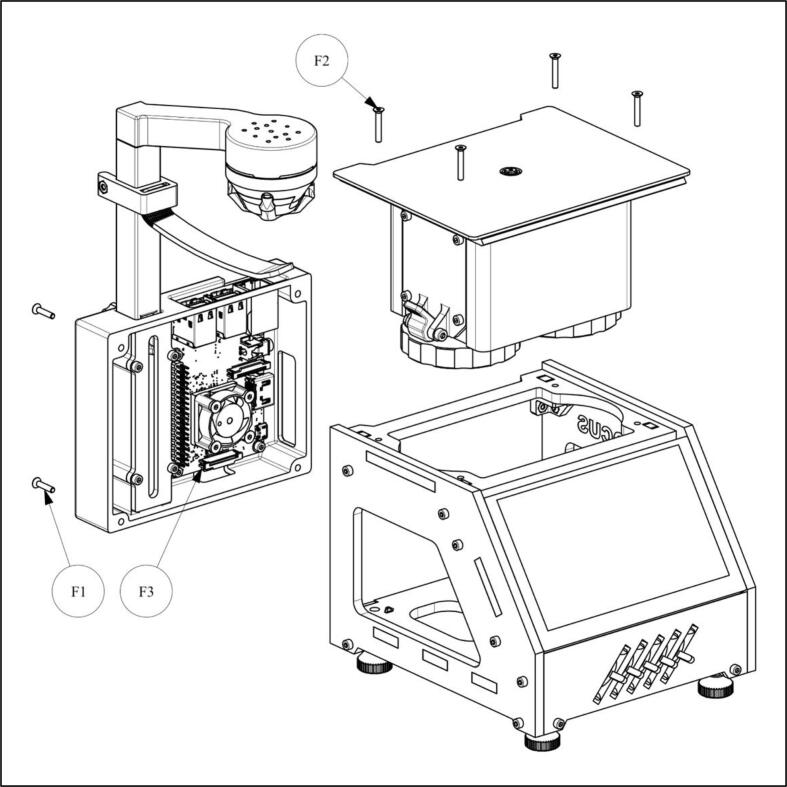
Schematic of final assembly.
2. Connect the ribbon cables (F3) to Raspberry Pi (EP4).
3. Fasten the electronic unit to the housing with four M2.5x12 countersunk screws (F1).

Disassembly is carried out in reverse order.

### 6.2 Apertures and filters

For samples with poor contrast, apertures and filters can enhance image contrast. Apertures for oblique and dark-field illumination are manufactured by sticking black adhesive foil onto a transparent foil, which is glued into the 3D printed “filter pan” (pos. H13). Filters for Rheinberg illumination are produced in a similar matter by sticking colored transparent foils onto a transparent foil, which is also glued into the 3D printed “filter pan” (pos. H13).

#### 6.2.1 Oblique illumination (Kreutz apertures)

1. Cut a transparent foil (A1) into a disc with 18  mm diameter. This is best done with a circle cutter.
2. Cut a black adhesive foil (A2) into a disc with 18  mm diameter.
3. Cut out a recess on one side such that the foil looks like a half-moon (see Fig. 4). We tested geometries with distances of 10  mm, 8  mm, and 6  mm at the widest point.
4. Carefully stick the adhesive foil (A2) onto the transparent foil (A1).
5. Glue the foil into the 3D printed “filter pan” (H13).

#### 6.2.2 Dark-field illumination

1. Cut a transparent foil (A1) into a disc with 18  mm diameter. This is best done with a circle cutter.
2. Cut a black adhesive foil (A2) into a small disc. We tested diameters of 8  mm, 10  mm, and 12  mm.
3. Carefully stick the adhesive foil (A2) into the center of the transparent foil (A1), see Fig. 4.
4. Glue the foil into the 3D printed “filter pan” (H13).

#### 6.2.3 Rheinberg illumination

1. Cut a transparent foil (A1) into a disc with 18  mm diameter. This is best done with a circle cutter.
2. Cut two colored adhesive foils of different colors (A3) into a discs and a ring (see Fig. 4). We used a dark color (green or blue) for the inner disc and a light color (orange, yellow, or red) for the outer ring. Die diameters of the inner discs were tested at 8  mm, 9  mm, and 10  mm.
3. Carefully stick the colored adhesive foils (A3) onto the transparent foil (A1).
4. We marked the interface of the two foils additionally with a black permanent marker.
5. Glue the foil into the 3D printed “filter pan” (H13).

### 6.3 Software

First, install a suitable Linux distribution according to the instructions on the Raspberry Pi website (*https://www.raspberrypi.com*). Please consult “6.6.3 Linux distributions” for tested images. Additionally, please note that you'll need to be logged in as root to install the software. To do so, open the Terminal and type: “sudo su”.

### 6.4 Raspberry Pi configuration

The Raspberry Pi configuration is not covered by the installer below, as it depends on the operating system used. Here are some recommendations:

#### 6.4.1 Raspi-config

The following settings should be manually set in “raspi-config.” Type “raspi-config” in the terminal. If a recent system is used (see Table 1), the legacy-camera support must be activated. Navigate to “3 Interface Options” > “I1 Legacy Camera” > “Enable”.

**Table 1:** OpenMicroView has been tested with the following Linux distributions.

**Image**	**Kernel**	**Firmware Hash**	**Comments**
raspios_oldstable_armhf 2019–09-26	4.19.75	01508e81ec1e918448227ca864616d56c430b46d	Passed
raspios_oldstable_armhf 2021–12-02	5.10.63	fa45ccf5a4b183ee566b36d74fb4b65bf9358bed	Passed
raspios_oldstable_armhf 2022–01-28	5.10.63	60f6a26ed5701eec6be5c864dd0db48fe6244fad	Passed
raspios_oldstable_armhf 2022–04-04	5.10.103	910e079df1266036159ce4ea2aaa2ba9976ea3f1	Passed
raspios_oldstable_armhf 2022–09-06	5.10.103	91e90da69cf0b1ddae23764b417bd6b43ec02c63	Passed
raspios_oldstable_armhf 2022–09-22	5.10.103	a17501d7c91a584085cd794ab9c007c9d1b9b435	Issues with screen and camera Not recommended
**raspios_oldstable_armhf** **2023**–**02-21**	**5.10.103**	**b57a33ad0991ffc19cd7b47cb7e20e3217705573**	**Passed** **Recommended**
raspios_oldstable_armhf 2023–05-03	5.10.103	638c7521ee0c431fafca1e2bd4fd25705b37ea5b	Issues with the touchscreen. Not recommended.
raspios_oldstable_armhf 2023–12-05	6.1.21	446f3…e19da	Issues with the touchscreen. Not recommended.

On older systems, the options are slightly different: “3 Interface Options” > “1 Camera” > “Yes”.

#### 6.4.2 Config file

The following settings should be set in the config file “/boot/config.txt”.

To edit the file, type “nano /boot/config.txt” in the terminal. Adjust the parameters as listed below, then save and exit the file (CTRL + S, CTRL + X).

**Table:** 

dtparam = audio = off	*Required for LED operations*
[all] start_x = 1 gpu_mem = 128	*Required for camera (automatically set with raspi-config)*

### 6.5 Installation of OpenMicroView

Download the software package from the publication repository, decompress it, and start the installation using the following command in the Raspberry Pi terminal:

**Table:** 

wget < url> && tar −xvz openMicroView_SW_v1.*.tar.gz && sudo OpenMicroView/install/install.sh −A

Replace < url > with the URL pointing to the software package. The link can be copied from the repository by right-clicking on the software package’s download button.

Alternatively, the latest available version of the software can also be downloaded and installed using the following command:

**Table:** 

git clone ssh://git@github.com/SpaceBiologyGroup/OpenMicroView −-depth 1 && sudo OpenMicroView/install/install.sh −A

### 6.6 General notes

#### 6.6.1 Permissions

The software has only been tested with root permission. If the software should run with normal user permission, connect the white LED wire to pin 19 (GPIO10) instead of pin 12 (GPIO18).

Then, edit src/open_micro_view/microscope_light.py, line 11:

LED_PIN = board.D10.

In the file “/boot/config.txt” the following configuration needs adjustment:

**Table:** 

dtparam = spi = on enable_uart = 1

Please note that running the process as a normal user has not been tested and may provoke permission errors while saving, reading, or copying pictures. Temperature readings may also not be available.

#### 6.6.2 Fan operation

If the fan is connected to the 5 VDC pin, it will always run as soon as the Raspberry Pi is powered, even after it has been shut down. If the fan should only run after a critical temperature has been reached, the fan can also be rewired to an alternative GPIO, such as pin 8 (GPIO14). Then, the settings in “raspi-config” must be adjusted. Type “raspi-config” in the Terminal. Navigate to “4 Performance” > “P4 Fan” > “Yes” −> Select the GPIO to which the fan is connected (e.g. 14) −> Define a threshold temperature (e.g. 60 °C) −>“OK.”.

#### 6.6.3 Linux distributions

The software has been developed and tested on a RaspberryPi 3B. The Linux distributions indicated in Table 1 have been tested with the software. System images can be downloaded from the official Raspberry Pi website: https://downloads.raspberrypi.com/raspios_oldstable_armhf/images.

## 7 Operation instructions

1. Power the “open*µ*View” microscope with the AC/DC converter supplied with the Raspberry Pi (Fig. 11). For mobile applications, a portable power bank supporting USB can be used alternatively.

**Fig. 11:**
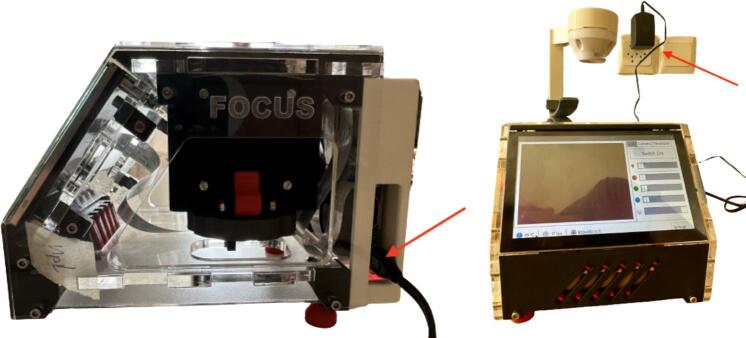
Connect the power supply to the power socket located at the “open*µ*View’s” far right corner (red arrow, left image) and power it (right image).
2. For optimal illumination, adjust the height of the light support. To slide the light support, loosen the knob at the rear side and tighten once the desired height has been reached (Fig. 12).

**Fig. 12:**
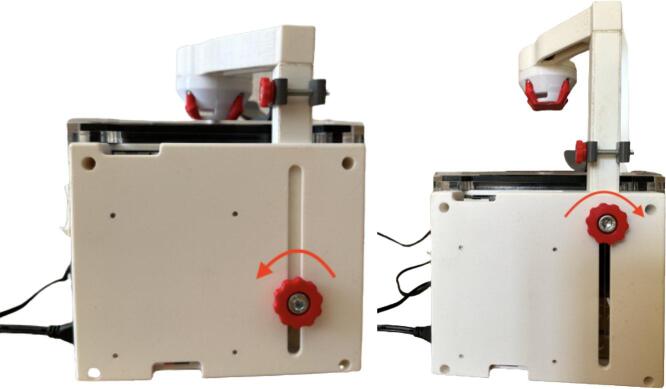
Loosen the knob counterclockwise (left image), adjust the height of the light support and tighten the knob clockwise (right image).
3. For poor-contrast samples, contrast can be enhanced by using an aperture or a colored foil in front of the LEDs. If desired, choose the aperture you want to work with and insert it in the filter holder or leave it empty (Fig. 13).

**Fig. 13:**
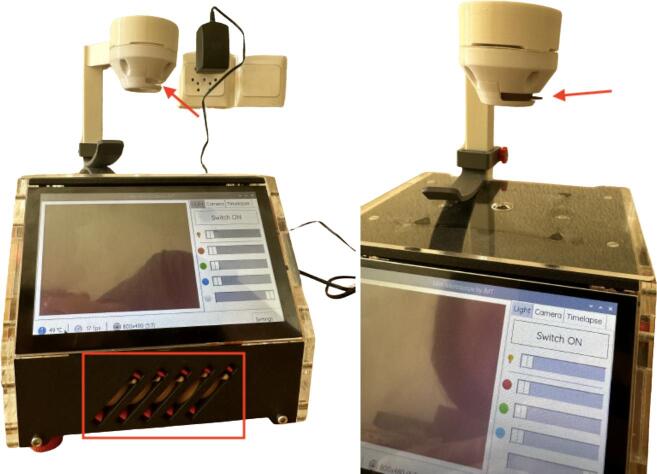
Contrast can be enhanced by placing an aperture or a colored foil in front of the LEDs (red arrow). Unused apertures and filters can be stored below the screen (red frame).
4. In the software, switch to the “Light” tab and press “Switch ON” to turn on the LEDs (Fig. 14).

**Fig. 14:**
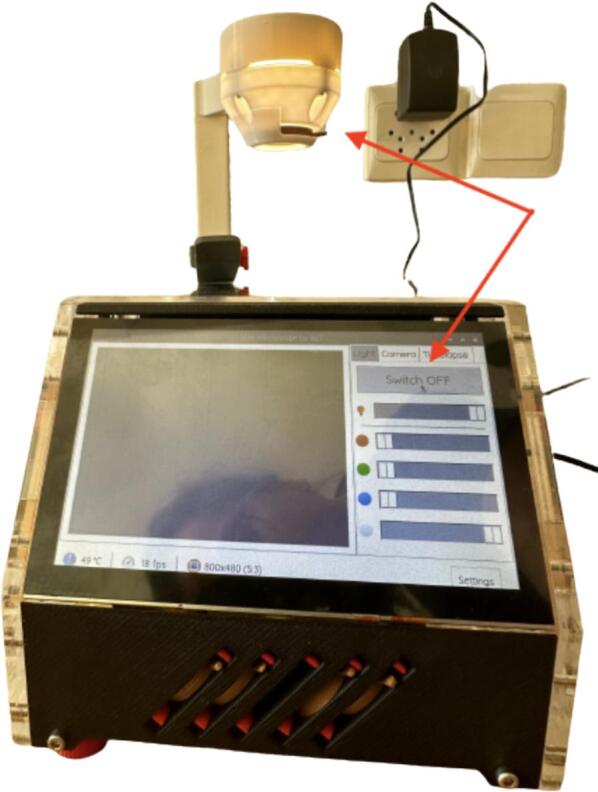
Use the “Switch ON/OFF” button on the “Light” tab to control illumination.
5. Place the sample on the sample stage. If desired, fix the sample with the leaf spring. Adjust the focus and zoom with the respective handwheel to get a focused image with the desired field of view.
6. To get a better image contrast, try adjusting the light settings or choose a different aperture. Adjusting the camera settings in the “Camera” tab may also help to improve results (Fig. 15).

**Fig. 15:**
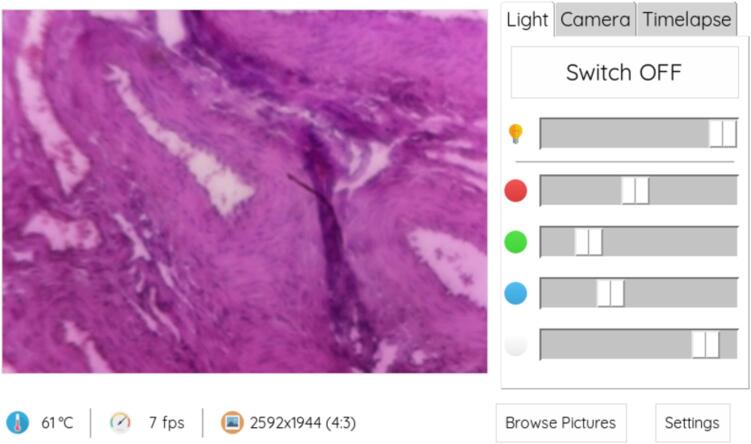
Adjusting the light settings in the “Light” tab may help improving image quality.
7. A still image can be acquired by clicking on the “Capture Image” button (Fig. 16). A time lapse can be acquired in the respective tab.

**Fig. 16:**
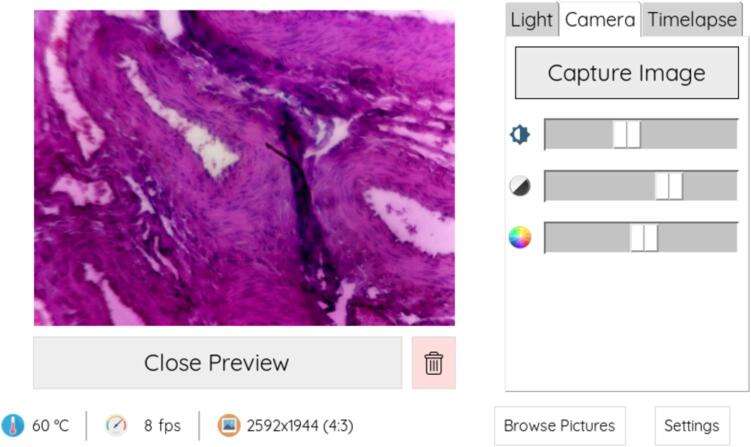
Adjusting the camera settings in the “Camera” tab may help improving image quality. Click on “Capture Image” to acquire a still image.

## 8 Validation and characterization

**Fig. 17:**
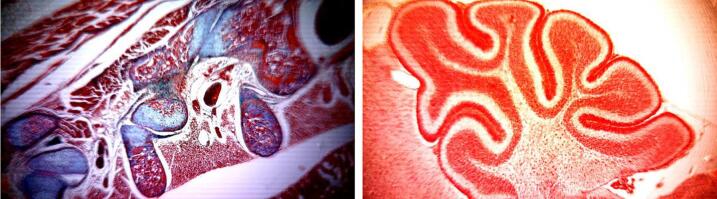
Histological samples of a rat embryo showing the spine (left) and the cerebellum (right). The images were taken from a commercially available sample (Johannes Lieder GmbH & Co., Germany).

**Fig. 18:**
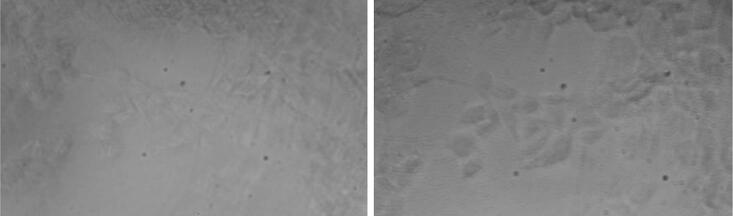
Contrast enhancement by oblique illumination (right) in comparison to brightfield illumination (left) on bovine chondrocyte culture.

**Fig. 19:**
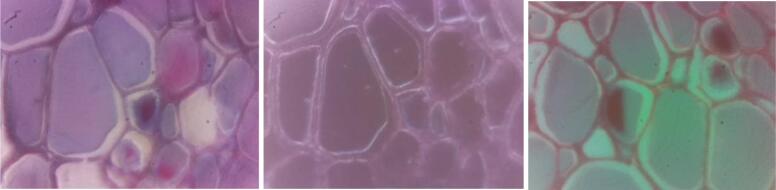
Brightfield (left) dark-field (center) and Rheinberg (right) illumination on ovine thyroid cells. For the Rheinberg illumination a central green and a peripheral orange filter was used. The images were taken from a stained and commercially available sample (Johannes Lieder GmbH & Co., Germany).

**Fig. 20:**
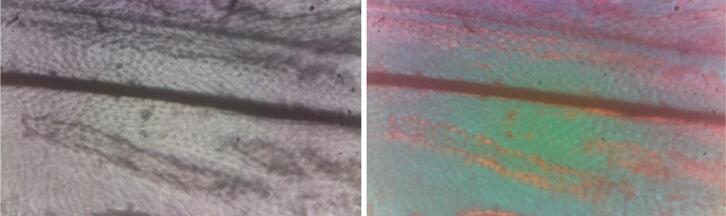
Rheinberg illumination on a fruit fly wing with a green central and an orange peripheral filter (right) in comparison to brightfield illumination (left).

Considering the microscope’s low cost in comparison to commercial lab microscopes, the acquired images are of surprisingly good quality (Fig. 17). Adjusting the magnification and focus can be tricky at the beginning and requires some practice. Generally, it is advisable to first focus at low magnifications and then start zooming into the region of interest while adjusting the focus as well. For samples with poor contrast in the brightfield, fine tuning the illumination (height, use of apertures, LED color, and brightness) or camera settings (brightness, contrast, and saturation) can make a significant difference. This also requires some experience and trial-and-error testing. For the different contrasting techniques, we recommend trying different aperture sizes and colors (Rheinberg illumination). Some achievable results are shown in Fig. 18 (oblique illumination), Fig. 19 (dark-field and Rheinberg illumination) and Fig. 20 (Rheinberg illumination). We most often achieved the best results using the white LEDs. Depending on the sample, adjusting the LED color could help to obtain a better image.

The software has no feedback about the position of the lens, camera and sample. Therefore, the resulting magnification is not known to the system. If scale bars or distance measurements are required, the image must be compared to a calibration target without moving the camera and lens. Also, currently the system does not handle lens shading and chromatic aberration. The aperture at the front end of the lens’ housing helps to avoid artifacts introduced by the lens’ peripheral part, which would result in a blurred image.

**Fig. 21:**
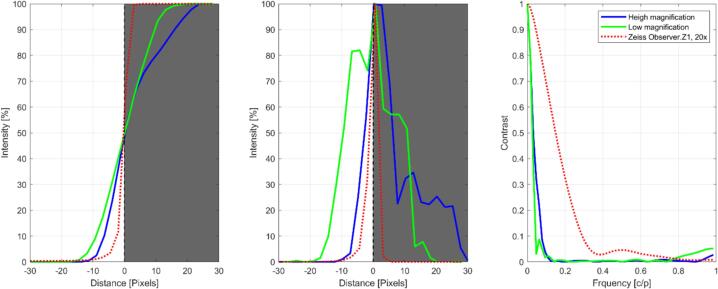
MTF analysis using a razor blade and the software “MTF Mappper” at a high magnification (blue lines), low magnification (green lines) and a Zeiss Observer.Z1 with 20x objective (red dashed lines, reference microscope). The edge spread function (ESF, left plot) indicates the intensity profile perpendicular to the edge, which would ideally be a step function. The line spread function (LSF, central plot) is the derivative of the ESP and ideally is an infinitely narrow vertical pulse. The spatial frequency response (SFR, right plot) is the Fourier transformed LSF. The ESP and LSF intensities were normalized to the respective maximum values for better comparability.

The achievable resolution and field of view partly depends on the thickness of the sample. We used an improved Neubauer counting chamber to examine which structures could still be resolved. At the highest magnification, we could still properly resolve 3  µm wide lines, with a field of view of ca. 400x300 µm. At the lowest magnification the field of view was ca. 2000x1500 µm, but the size of still resolvable lines increased to ca. 10  µm. With lower magnification it becomes more difficult to still achieve homogenous illumination, making the peripheral areas appear darker than the center (e.g. Fig. 17). Bringing the LEDs closer to the sample can help getting a better result, especially in brightfield illumination (without contrasting techniques). In some samples we observed that the peripheral areas were out of focus (e.g. Fig. 17). However, we did not see it with the Neubauer counting chamber, which suggests that this artifact could be related to the flatness of the sample. Finally, we did some rudimentary slant edge modulation transfer function (MTF) tests using a razor blade and the software “MTF Mappper” (v0.7.38) [13]. The MTF analysis is a resolution performance test, which examines how well the optical setup can resolve a straight edge with a dark and bright side. We performed the test at a high and a low magnification to capture both extremes. (The camera settings were set to reach a maximal contrast.) As a reference the same test was also performed using the high-end microscope Zeiss Observer.Z1 (20x, NA 0.4 objective). The results show the limitations of our approach (Fig. 21). The edge spread functions (ESF), which indicate the intensity profiles perpendicular to the edge, are not as steep as compared to the reference microscope. Consequently, the line spread functions (LSF), which are the derivatives of the ESPs, are more spread out. The spatial frequency response (SFR), which is the Fourier transformed LSF, shows the reduced contrast at high frequencies. The low-cost lens is likely the main reason for these limitations. Therefore, other designs preferred high quality objectives [7].

Currently the software is solely intended for image acquisition and temporary image storage. The Raspberry Pi’s computational power was sufficient for these simple tasks. In future software upgrades, simple image processing with low computational requirements is conceivable. However, tasks that are heavy on computational power or require large memories or large data sets (e.g. AI training data) will not be feasible. Such tasks could be outsourced to a webservice, which the Raspberry Pi could be linked to.

In conclusion, here we presented a simple, single-lens microscope that leverages a Raspberry Pi with camera. In our research, we use it to check cell culture in temporary field labs. However, the microscope is also suitable for other applications, including educational and non-professional use.

## 9 Declaration

**Ethical statement**.

All biological samples have either been purchased commercially or did not require ethical permission.

## Credit authorship contribution statement

**Vincent Salvadori:** Software. **Daniel Fäh:** Methodology. **Sarina Flühler:** Methodology. **Jan Wandeler:** Methodology. **Maria J. Jacome:** Writing – original draft. **Adrian Koller:** Supervision. **Marcel Egli:** Supervision. **Simon L. Wuest:** Writing – original draft, Software, Conceptualization.

## Declaration of competing interest

The authors declare that they have no known competing financial interests or personal relationships that could have appeared to influence the work reported in this paper.
